# Kratom-Induced Neurovasculitis: A Case Report

**DOI:** 10.51894/001c.165687

**Published:** 2026-07-31

**Authors:** Khalie Mahoney, Reagan Mahoney, Joe Churukian

**Affiliations:** 1 Henry Ford - Warren

## 96

### Background

Kratom, the products derived from the leaves of Mitragyna speciosa, are rapidly becoming more clinically relevant as more patients are using it both for intoxication and as a means of bypassing withdrawal symptoms from other substances. It is legal for sale and consumption in much of the United States but is not FDA approved for medical treatment. At low doses, it appears to have stimulant effects; at higher doses, it has more sedative/opioid action. Known side effects include rhabdomyolysis, seizures, nonfocal neuropathy, and hepatotoxicity.

### Case description

Patient NB is a 29 year old female presenting to the ICU from behavioural health for suspected seizure without a prior history of epilepsy. She was found having generalized convulsions and was nonverbal. The patient was given a constant infusion of benzodiazepines and transferred to the ICU. Labs showed a high anion gap acidosis and a high CPK. With the concern for possible encephalitis patient was given antibiotics and acyclovir. CT was unremarkable but EEG showed paroxysmal runs of theta waves localized to the right frontotemporal region. Patient was able to be weaned off of Versed and began answering questions, increasingly aware and able to interact with her environment. MRI of the brain was ordered and patient was found to have a “small focus of signal abnormality and restricted diffusion within the splenium of corpus callosum.” Her stepfather informed us of her high dose Kratom use for her recovery from polysubstance abuse. Repeat EEG the following day showed improvement in the theta activity, and patient was downgraded from the ICU.

### Discussion

As stated by the radiologist on the MRI report, the lesion likely represented a reversible splenial lesion likely in the setting of drugs/toxins, seizures, antiseizure medications, metabolic disturbances, etc. The neurologist narrowed his diagnosis to kratom-induced vasculitis with a small stroke or encephalitis. NB and the neurologist had multiple discussions about her need to avoid kratom going forward. As more people begin turning to kratom, it will be increasingly important to have a more complete picture of the adverse effect profile as well as medication interactions.

